# Scale-Up of a Rh-Catalyzed Asymmetric sp^3^–sp^2^ Suzuki–Miyaura-Type Reaction

**DOI:** 10.1021/acs.oprd.2c00268

**Published:** 2022-11-02

**Authors:** Laura Cunningham, Mireia Sidera Portela, Stephen P. Fletcher

**Affiliations:** † Department of Chemistry, Chemistry Research Laboratory, University of Oxford, Oxford OX1 3TA, U.K.; ‡ Vertex Pharmaceuticals (Europe) Ltd., Abingdon OX14 4RY, U.K.

**Keywords:** asymmetric catalysis, sp^2^−sp^3^ cross-coupling, Suzuki−Miyaura stereogenic centers, enantioconvergent, scale-up

## Abstract

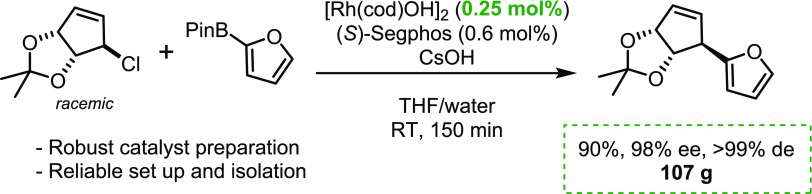

Csp^2^–Csp^2^ Suzuki–Miyaura couplings (SMCs) are ubiquitous in the synthesis of small molecules, but analogous Csp^2^–Csp^3^ bond-forming SMCs are rare, especially asymmetric variants. Recently, we developed a series of Rh-catalyzed couplings between racemic sp^3^-hybridized allyl chlorides and heteroaryl boronic acids. Here, we demonstrate that these catalytic asymmetric reactions can be scaled-up to give over 100 g of a product. The reaction we chose to test couples a heteroaromatic boronic acid derivative and a racemic bicyclic electrophile to give a product with three contiguous stereogenic centers. The SMC product was obtained as a single diastereomer in 90% yield and 98% ee. Kinetic analysis of the reaction reveals two exothermic steps in the reaction setup and revealed the means by which to prevent the generation of heat spikes detrimental to the stability of the catalyst.

## Introduction

Reactions that form carbon–carbon bonds are central to organic synthesis.^1−3^ One of the most widely used carbon–carbon bond-forming reactions, particularly in pharmaceutical research and manufacturing, is Suzuki–Miyaura coupling (SMC), which is used in about 25% of all medicinal chemistry publications.^4^ Many sp^2^-hybridized boronic acids are stable and readily available coupling partners, helping to make SMC a robust and general methodology that is globally employed. SMC provides tremendous advantages over procedures that employ highly reactive, air- and moisture-sensitive organometallic reagents^5^ and has been used as the key step in many large-scale processes.^2,6−9^

**Figure 1:**
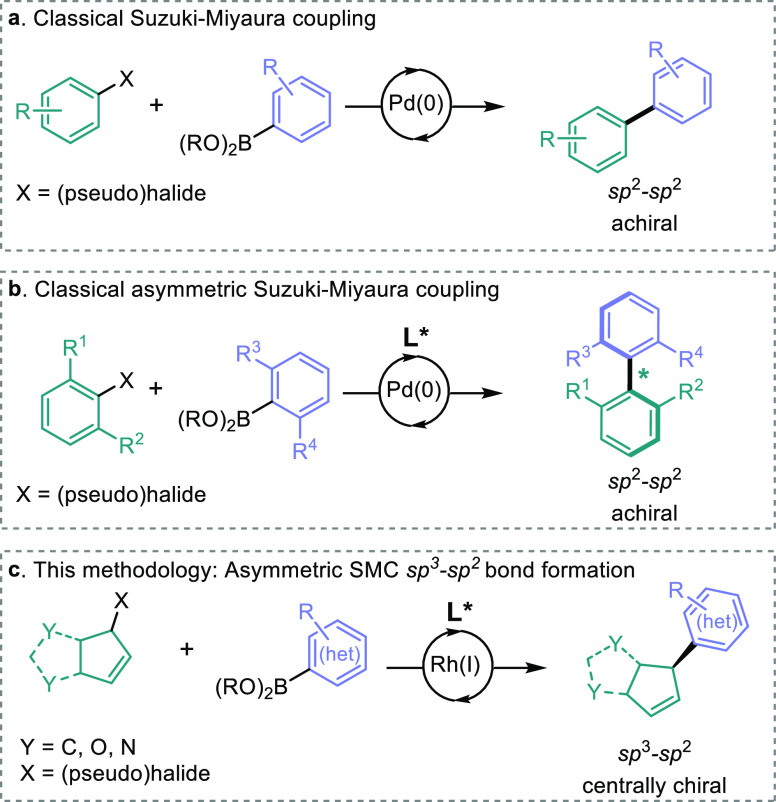
(a) Classical SMC to give achiral biphenyl motifs. (b) SMC of two sterically demanding sp^2^-hybridized substrates to give axially chiral products. (c) Rh-catalyzed asymmetric SMC of racemic sp^3^-hybridized allyl halides and boronic acids/esters.

The ease with which SMC enables sp^2^–sp^2^ coupling has been reported to have led to planar molecules dominating drug discovery programs (Figure 1a) so that as much as 75% of all species in fragment-based drug discovery libraries are one- or two-dimensional in shape.^10^ This is despite the knowledge that as drug candidates pass from discovery to commercial drugs, the density of sp^3^-carbons and number of chiral centers increase, highlighting the need for methodologies that are as practically simple for the synthesis of three-dimensional molecules.^11−13^ Enantioselective sp^2^–sp^2^ coupling methods for the synthesis of axially chiral biaryls have been developed, which do enable access to a specific class of three-dimensional compounds (Figure 1b).^14−18^

The development of systems that can exploit the generality and practical simplicity of SMCs yet yield enantiomerically enriched three-dimensional products with sp^3^ centers has been a major research goal for many years.^17,18^ Csp^3^-hybridized coupling partners are rare in catalytic asymmetric SMCs, but some examples using enantiomerically enriched Csp^3^ coupling partners as well as secondary halides and asymmetric addition to *meso*-compounds have been reported.^19−24^ These asymmetric methods are generally limited by the use of specific nucleophiles or electrophiles and simple aliphatic and aromatic substrates, lacking the functionality inherent in medicinal compounds. Methods which tolerate heterocycles in asymmetric SMCs are still lacking despite their prominence in biologically active molecules and this topic continues to be one of active research.^25−27^ While we have developed methods aimed at beginning to address these issues (Figure 1c), they have yet to be demonstrated on a scale useful for industrial processes.

**Scheme 1:**
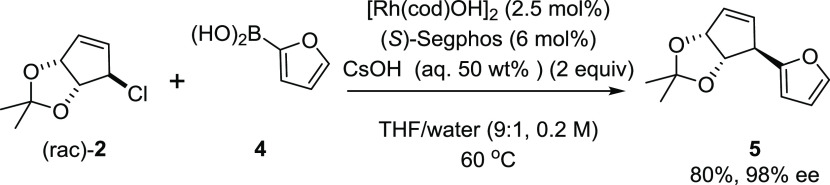
Initially Examined Conditions for the Synthesis of Allyl Furan **5** Reaction carried out on 200 mg of allyl chloride.

**Figure 2:**
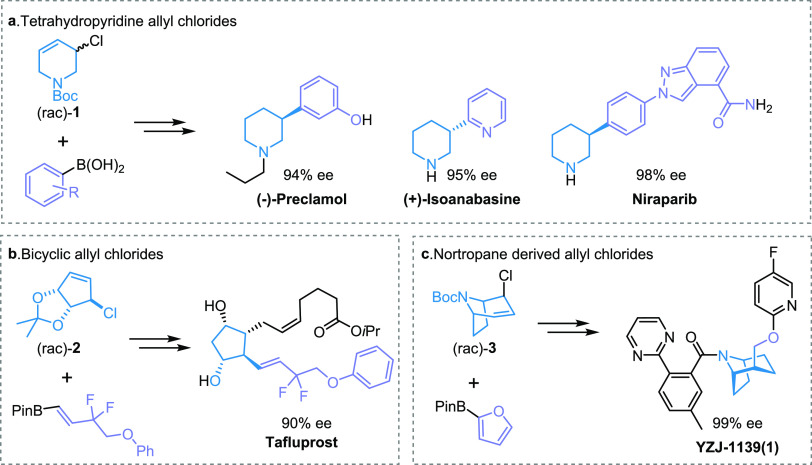
(a) Catalytic asymmetric synthesis of drug molecules from tetrahydropyridine allyl chlorides using a key Suzuki–Miyaura-type coupling. (b) Total synthesis of a prostaglandin analogue from bicylic allyl chlorides. (c) Formal synthesis of a potential insomnia drug in phase II clinical trials from nortropane-derived allyl chlorides. In all cases, the ee’s shown were achieved during the key Suzuki–Miyaura-type coupling step.

We have reported several enantioselective SMCs between (hetero)aromatic boronic acids and racemic sp^3^ coupling partners (Figure 2).^28−33^ This methodology is related to Tsuji–Trost allylation, which typically uses Pd catalysts and stabilized nucleophiles.^34,35^ The electrophilic sp^3^ coupling partners are racemic allyl halides of 5–7 membered rings that are pseudo-symmetric about the allyl halide unit, allowing catalyst-controlled enantioconvergent reactions. The allyl halides may be all-carbon or contain heteroatoms, such as tetrahydropyridines, and bicyclic systems, including nortropane derivatives, are also suitable substrates. These allyl halides are derived from allyl alcohols. Numerous allyl alcohols are commercially available, including some which have proven to be suitable scaffolds for this methodology.^30,31^ More complex allyl chlorides we have accessed can be obtained in a few steps from bulk building blocks such as pyridine (Figure 2a) or cyclopentadiene (Figure 2b). Diverse targets are accessible with these methods as illustrated by the stereocontrolled synthesis of the biologically active molecules shown in Figure 2.^33,36,37^

As of yet, there have been no reported investigations of the scalability of these reactions beyond a gram scale—a key factor that needs to be demonstrated if these methods are to be used in process chemistry. Here, we seek to address the applicability of these reactions on scales relevant to industrial chemistry by demonstrating this cross-coupling chemistry on a far greater scale than has previously been reported. We show that catalytic asymmetric stereogenic center forming SMC-type reactions can give 100 g of a complex product in an academic environment, suggesting that this methodology may be suitable for process applications.

## Discussion

Investigating if asymmetric SMC reactions are suitable for process chemistry requires exploring several factors, including (1) the scalability of the chemistry, (2) understanding potential hazards, and (3) minimizing the catalyst loading. We also felt it was important to examine the chemistry on a nontrivial substrate, and so carefully considered which reaction to perform. Many of our early examples were done using relatively simple all-carbon electrophiles and benzene-derived boronic acids, and although these reactions work well and have readily available starting materials, we felt that it was important to try reactions with substrates which would be more relevant to real-life applications. Additionally, some targets, which we have previously made, such as niraparib, can also be accessed by asymmetric hydrogenation or enzyme-mediated transformation,^38^ and we considered the value in making products that could not easily be produced by other means. We identified allyl furan **5** as a suitable candidate (Scheme 1).

Allyl chloride **2** enables the setting of three contiguous stereocenters in a single enantioselective and diastereospecific transformation. Manipulation of the cyclopentene core and geminal diol can easily give rise to a range of complex derivatives, as we have previously shown.^37^ 2-Furan boronic acid was selected as heteroaryl motifs are significantly more challenging than phenyl-based systems, and furans are present in multiple commercial medicines such as mometasone and nitrofurantoin.^26,39^ In our own studies, 2-furan nucleophiles have proven to be challenging to employ with high efficiency,^31,33,36^ and so we reasoned that if we could optimize this particular substrate, this method would likely be compatible with a wide range of aryl- and heteroaryl nucleophiles. Furan derivatives are also attractive as they serve as a powerful synthetic handle and can be converted to yield highly oxygenated species and other heteroaryls and carboxylic acids.

A set of conditions were identified which gave access to **5** in excellent selectivity (98% ee) and 80% yield, on a 200 mg scale (Scheme 1).^29^ A target scale of 100 g was set, which was deemed to be large enough to indicate suitability to even larger-scale applications as we would be able to detect exotherms and deleterious macroscopic physical effects associated with mixing and phase behavior. A secondary target was to reduce the catalyst loading by a factor of 10 vs our standard conditions to reduce the relative cost of the catalyst and the product. The initial procedure for this reaction involved adding the ligand to the metal, and the resulting complex was subsequently added to a solution containing both coupling partners, followed by the addition of a base as the final reagent.^29^

### Reproducibility and Complexation Study

**Scheme 2:**
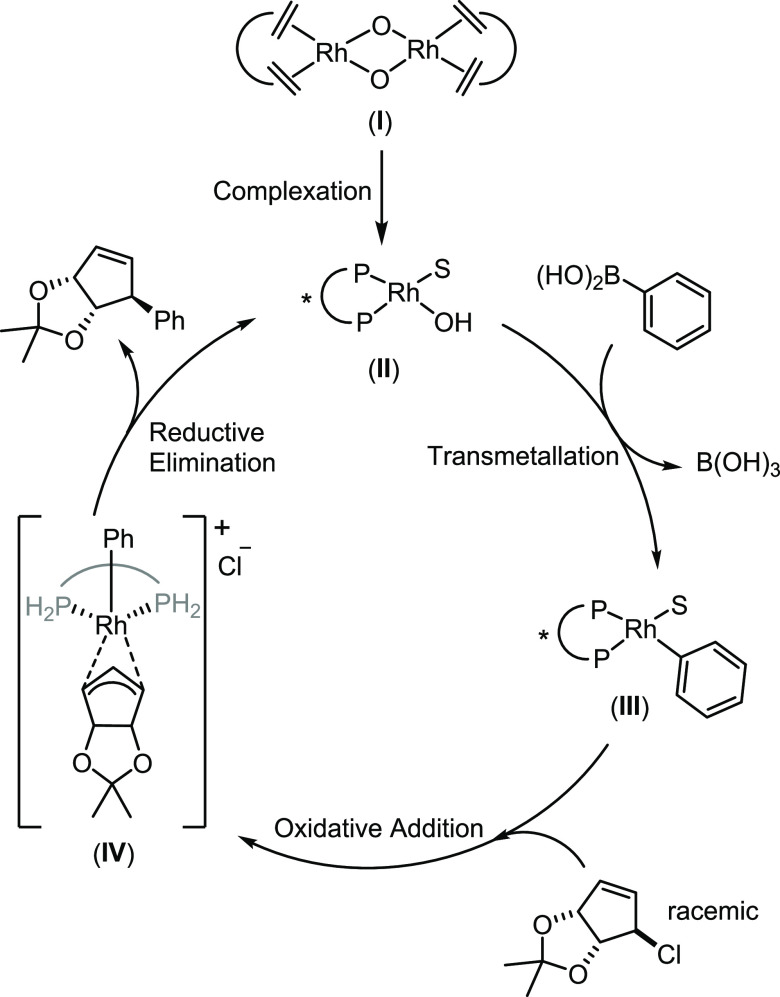
Mechanism of Reaction. S = Solvent

Initial attempts of reducing the catalyst loading and scaling up the reaction from 200 mg to only 1 g gave considerable reproducibility issues, with conversion varying by up to 40% in seemingly identical side-by-side reactions. While 70% was the most commonly observed yield, the conversion varied from 50% to 90%; however, 98% ee was consistently observed and the *cis* diastereoisomer was never detected. Product **5** is stable and leaving the reaction overnight did not appear to affect the yield. The key mechanistic steps in this transformation (Scheme 2) are reasonably well understood and help to understand potential sources of irreproducibility.^40^

We first sought to understand the efficiency of preparing the metal–ligand complex **II**. ^31^P NMR spectroscopic analysis revealed that the conditions used to form **II** (30 min at 60 °C with [Rh(cod)OH]_2_**I** and ligand in tetrahydrofuran (THF)) resulted in incomplete formation of **II** and the generation of a variety of additional species, which were formed in unpredictable amounts (Supporting Information Figure S1). While any effect of the poorly selective catalyst complex formation appears to be negligible on typical methodology development and substrate scope scales, which uses 5 mol % of the catalyst complex, given our interest in reducing the catalyst loading, we wanted to cleanly and reproducibly generate **II**.

Replacing [Rh(cod)OH]_2_ with [Rh(C_2_H_4_)Cl]_2_ gave fewer side products, yielding significantly cleaner spectra, likely due to the increased lability of ethylene vs cyclooctadiene. While complexation at 60 °C with [Rh(C_2_H_4_)Cl]_2_ is cleaner than with [Rh(cod)OH]_2_, there are still small amounts of unwanted metal–ligand complexes observable by ^31^P NMR spectroscopy. For this reason, complex formation at room temperature is preferred, which selectively generates **II** alongside small amounts of ligand-oxide (see Figure S2).

Transmetallation of **II** with boronic acid to give **III** proceeds cleanly at room temperature (see Figure S3a). However, upon heating the transmetallation complex **III** to the typical reaction temperature of 60 °C, degradation could clearly be observed by ^31^P NMR spectroscopy within 30 min (see Fig S3b). We found that performing SMCs at 40 °C did not lead to decomposition of the complex, enantioselectivity, or reaction time on a 1 g scale. We note that when using 2.5% [Rh(C_2_H_4_)Cl]_2_ or [Rh(cod)OH]_2_, the observed impurities appear irrelevant, but they are likely to significantly impact reactions at lower loadings. With this new complexation regime, the reproducibility of reactions performed on a small scale (∼1 g) at 40 °C improved considerably, and we consistently observed 80% conversion and 98% ee.

We next sought to increase the reaction concentration; while 0.2 M is suitable for examining substrate scope, it is not ideal for larger scales. Increasing the concentration to 0.9 M (1.6 g/10 mL) had no measurable impact on the conversion or enantioselectivity and would allow for a reasonable volume of solvent (650 mL) at the target scale.

**Figure 3:**
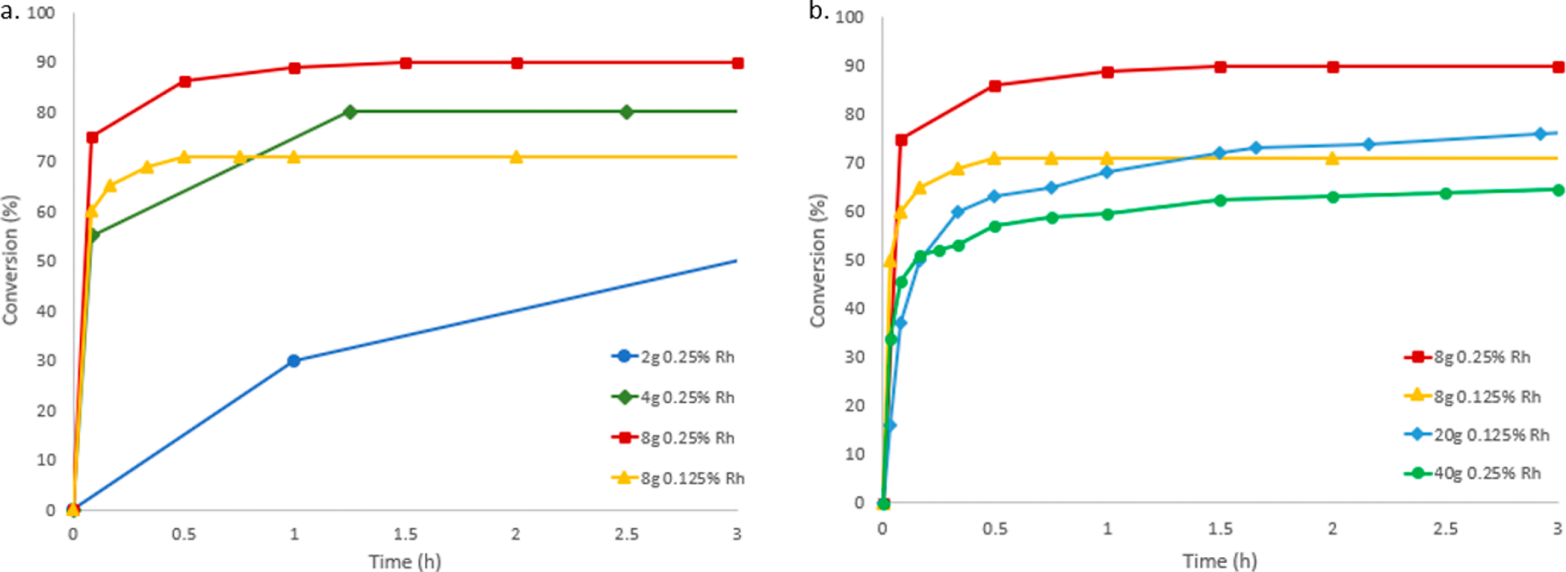
(a) Comparing conversion vs time in 2, 4, and 8 g scale reactions using either 0.125 or 0.25% [Rh(C_2_H_4_)Cl]_2_. (b) Comparing conversion vs time in 8, 20, and 40 g scale reactions.

The catalyst loading was investigated and we found that we could use 0.25% [Rh(C_2_H_4_)Cl]_2_ on a 1 g scale. Following reaction progression by quantitative ^1^H NMR spectroscopy showed that the initial reaction rate was rapid and that conversion appeared to increase with scale when going from 2 to 4 g and then 8 g reactions with 0.25% [Rh(C_2_H_4_)Cl]_2_ (Figure 3a). Upon reducing the loading to 0.125% [Rh(C_2_H_4_)Cl]_2_ on an 8 g scale, the conversion decreased markedly. We considered whether the trend of increasing scale giving rise to increased conversion would continue by carrying out a 20 g reaction using 0.125% [Rh(C_2_H_4_)Cl]_2_, but the conversion did not increase. This was initially hoped to be a consequence of the reduced catalyst loading, but returning to 0.25% [Rh(C_2_H_4_)Cl]_2_ for a 40 g reaction did not ameliorate the stunted conversion (Figure 3b). At this point, there was no obvious trend that related conversion to either scale or catalyst loading.

### Proposed Issues with Scale-Up

We considered two probable causes for the lower conversions observed at larger scales:

(A) Catalyst degradation: Temperatures of 60 °C were shown to cause decomposition. To reduce protodeborylation,^41^ CsOH is the last reagent added, after the catalyst. However, an exothermic reaction between the boronic acid and CsOH may generate sufficient heat to degrade the catalyst.
(B) Phase separation: As this is a biphasic reaction, reaction rate may be limited by the interfacial surface area. We have seen that the boronic acid, upon reaction with a base, partitions almost exclusively into the aqueous layer. As the reaction scale increases, the volume to interface area ratio increases, which may—in combination with phase effects—reduce conversion.

We carried out a series of reactions where we recorded internal temperatures using 0.125% [Rh(C_2_H_4_)Cl]_2_. Unsurprisingly, a significant increase in temperature was observed when the base was added (SI Figure S4), and so we opted to add the base at RT, before placing the reaction flask in an oil bath and then adding the catalyst. However, the addition of the catalyst itself led to similar temperature spikes, which we attributed to the heat of the SMC (SI figure S5). To keep the internal reaction temperature below 40 °C and to avoid catalyst decomposition, a reaction was carried out at room temperature. Gratifyingly, the conversion (76%) and ee (98% ee) were unaffected, and a temperature spike of only 5 °C was observed (SI Figure S6). All subsequent reactions were carried out with a 25 °C oil bath.

**Figure 4:**
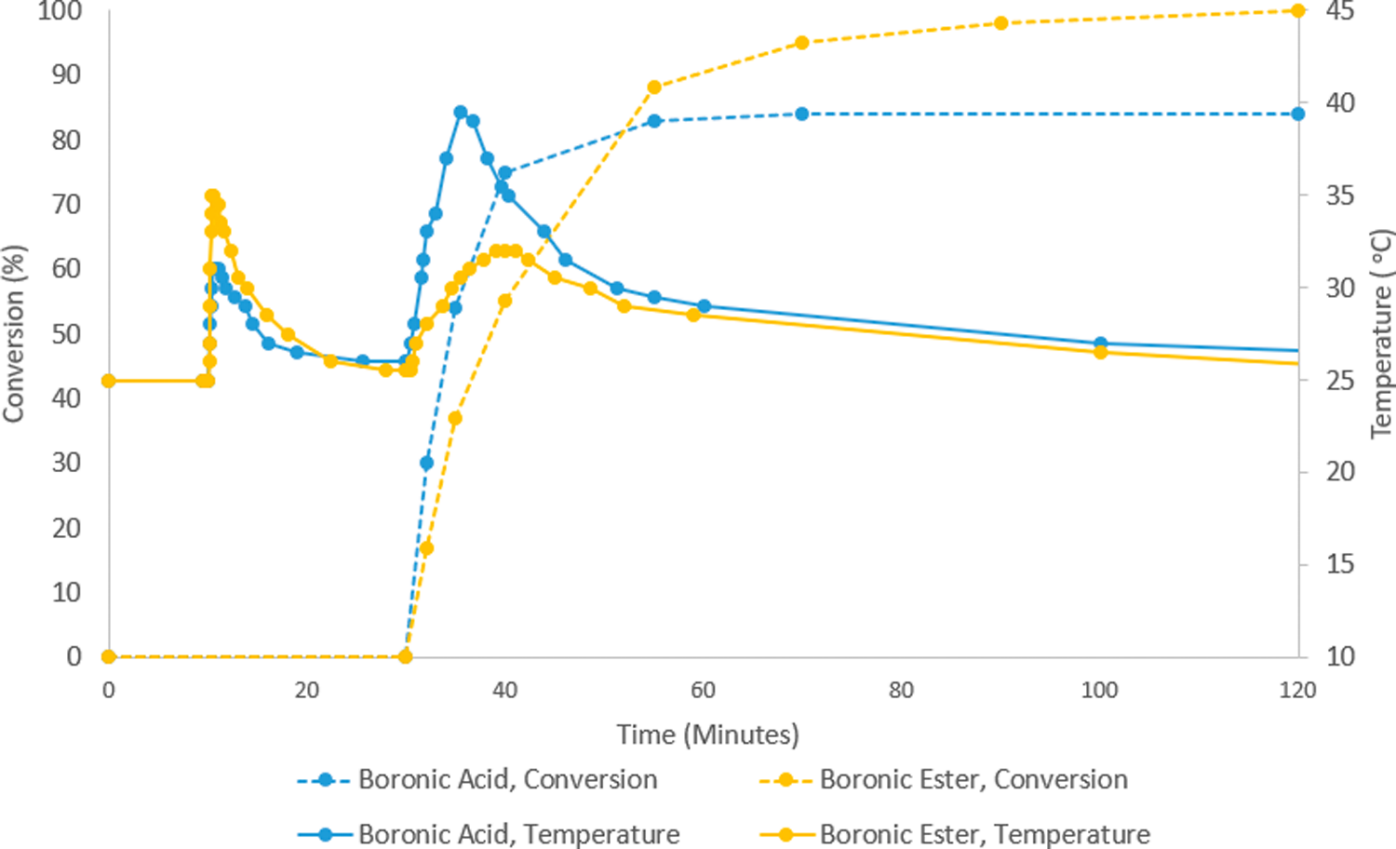
Temperature profile comparison of boronic acid (blue) and boronic ester (yellow). Reactions carried out on a 4 g (23 mmol) scale. CsOH added at 10 min. Catalyst added at 30 min.

Given the apparent heat generated by the SMC, we examined the boronic ester; boronic esters have been hypothesized to slowly release boronic acid, which then undergoes transmetallation.^42,43^ Using the pinacol ester did indeed significantly improve the conversion and appeared to ameliorate the issues discussed *vide supra*. The temperature increase upon catalyst addition was approximately halved when using the boronic ester vs the boronic acid (Figure 4). A significant change in phase behavior was observed when using the boronic ester. While the THF–water solution immediately separates into two layers when the base and boronic acid are combined, using the boronic ester does not lead to a biphasic mixture until ∼70% conversion. The late-stage phase separation is likely due to generation of CsCl byproduct, which is easily visible, salting-out the aqueous layer. Notably, despite the formation of a biphasic mixture at high conversion, significant quantities of boronic ester are still detectable in the organic layer. By comparison, when furan-2-boronic acid was employed, only trace amounts of heteroaryl-B(OH)_2_ were observed in the organic layer by ^1^H NMR spectroscopy immediately upon the addition of CsOH, supporting the idea that the biphasic system may be detrimental when using boronic acids.

**Figure 5:**
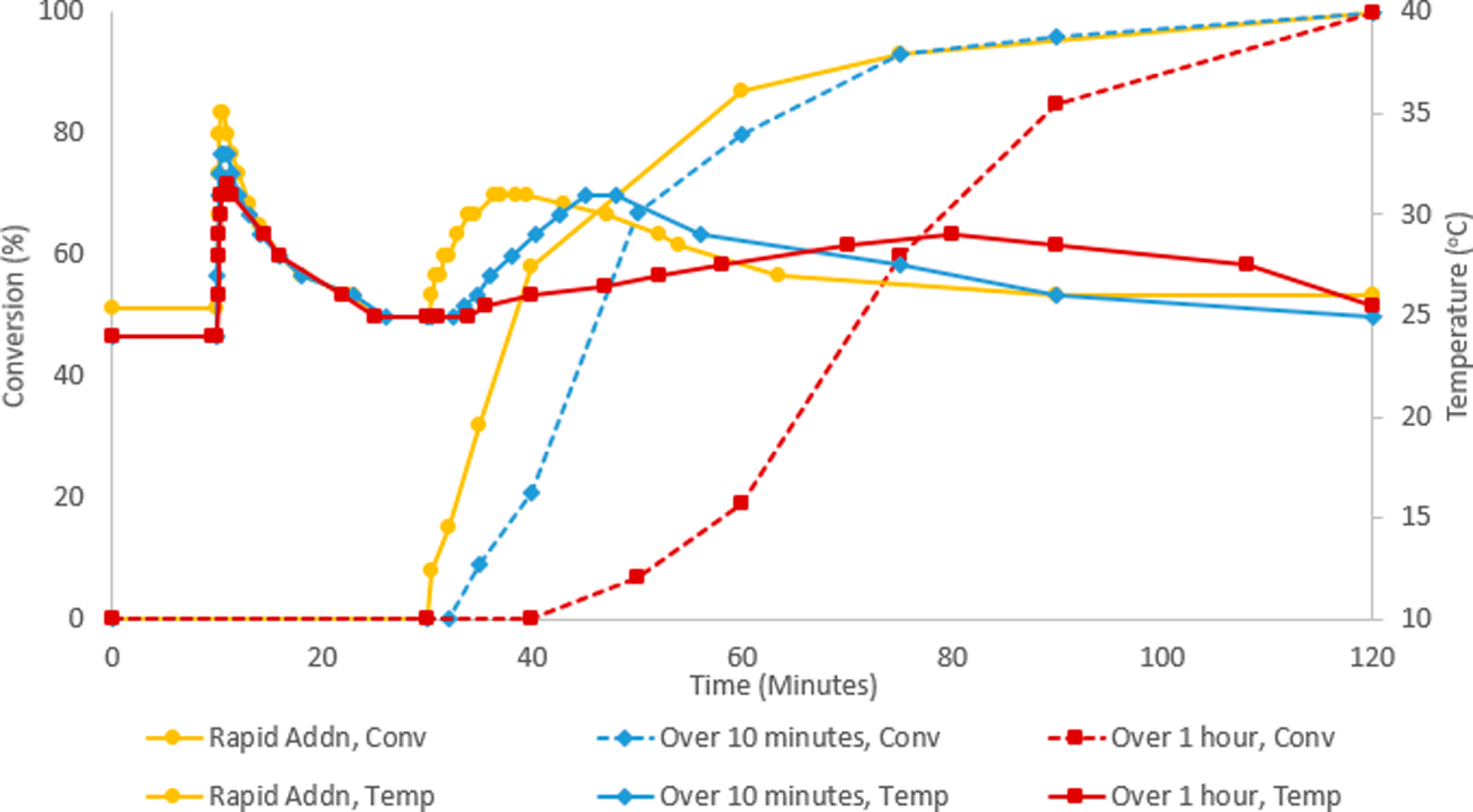
Comparison of internal temperatures and conversion between normal and slow addition of the catalyst. Base addition at 10 min over 10 s, and catalyst addition at 30 min over 10 s (yellow), 10 min (blue), or 1 h (red). Reactions carried out on a 4 g (23 mmol) scale of allyl chloride.

To further reduce the heat spike, slow addition of the catalyst solution was carried out. Although the initial rate of reaction was slower when the catalyst was added via a syringe pump over 10 or 60 min, slow addition of the catalyst did not affect the temperature maximum when added over 10 min; it simply delayed it. When addition was carried out over an hour, only a minor decrease to the maximum internal temperature was observed (6 vs 4 °C, Figure 5), which was considered unlikely to allow sufficient control of heat release at larger scales without prolonging the reaction time significantly. In all cases, complete conversion was observed 90 min after catalyst addition. While suitable control may be possible by carrying out slow addition of the catalyst over much longer time scales, we sought alternative resolutions that would not require considerable lengthening of the reaction time.

**Figure 6:**
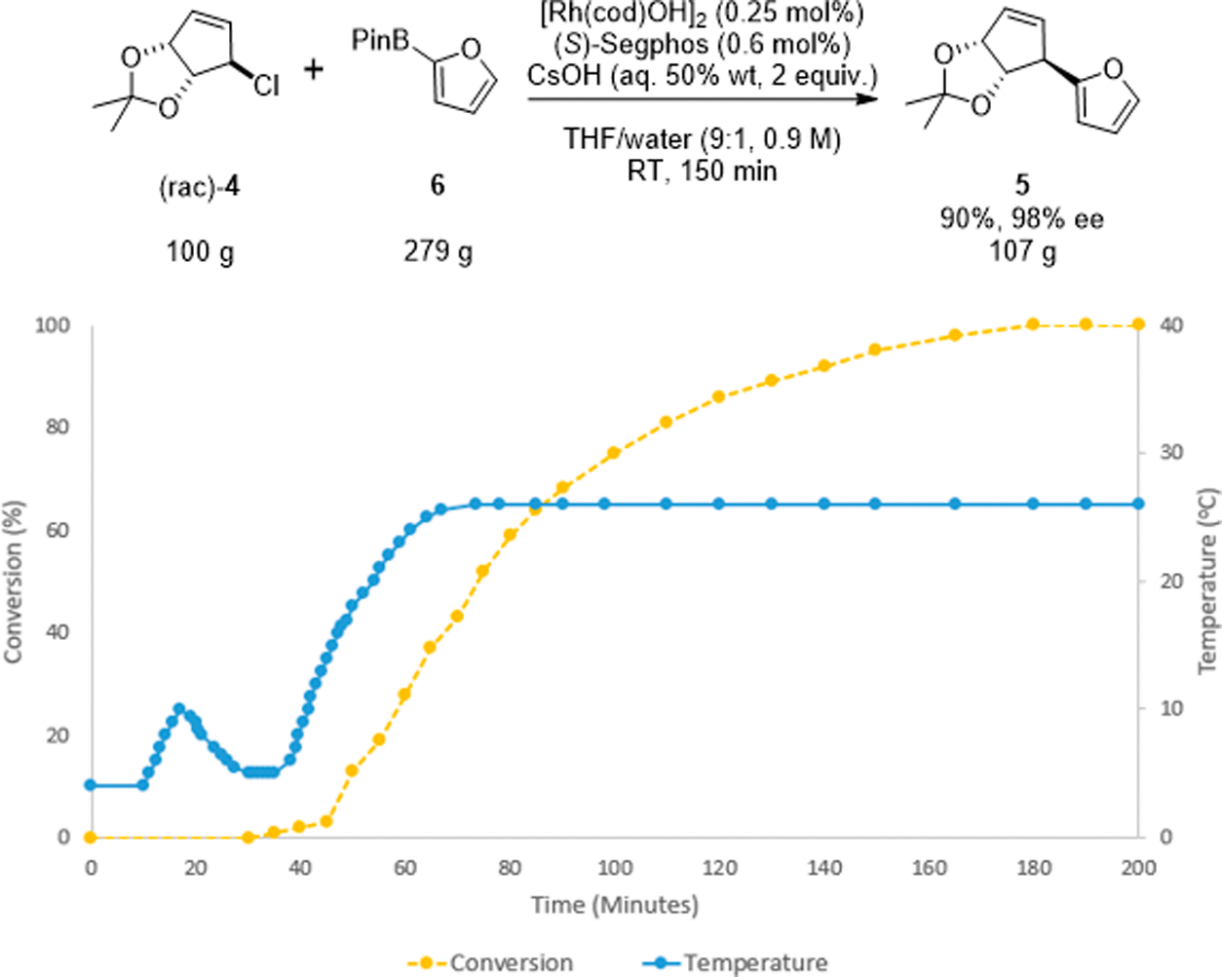
Final conditions for target reaction: internal temperature and conversion of 100 g scale reaction (0.65 mol allyl chloride). Allyl chloride, boronic ester, and solvent in ice bath. CsOH added over 3 min, starting at 10 min. The catalyst complex added over 3 min, starting at 30 min. At 35 min, the ice bath was replaced by a 20 °C bath.

We sought to avoid >30 °C internal temperatures and so examined reactions carried out at 0 °C. It was found that conversion is minimal at <5 °C, and so 5 min after addition of the catalyst, the ice bath is replaced with a 25 °C oil bath. Once the internal temperature reaches ∼15 °C, the rate of the reaction greatly increases (Figure 6). After further investigation, it was found that by replacing the ice bath with a bath at 20 °C, it was possible to prevent internal temperatures exceeding 26 °C while still enabling a fast reaction rate (Figure 6).

The required purification method depends on whether the boronic acid or ester is used. When the boronic acid was employed, protodeborylation generated furan, which is simply removed by evaporation, and allowed facile product purification by distillation. However, the relative stability of the boronic ester led to a more challenging purification. The temperatures required for distillation appear to hydrolyze the pinacol ester, leading to evaporation of pinacol, which solidifies in the distillation apparatus. To hydrolyze the ester prior to distillation, we found that after celite filtration to remove CsCl from the reaction mixture, stirring the crude product with 3M NaOH at 45 °C completely hydrolyzed the furan boronic ester, liberating furan and pinacol. After repeated washing with water to remove the pinacol, and evaporation to remove the furan, distillation yielded 107 g of pure product (90% yield, 98% ee).

## Conclusions

Here, we described the scale-up of an asymmetric Suzuki–Miyaura-type coupling between a racemic sp^3^-hybridized electrophile and heteroaryl boronic ester. Two exothermic processes have been identified: the addition of a base and addition of a catalyst, and we show that the release of heat can be easily controlled. The catalyst loading was reduced to 0.25% [Rh(C_2_H_4_)Cl]_2_, 10 times less than which is normally reported for this methodology, without any detriment to yield or enantioselectivity, and we suspect that it could be further lowered if desired. A 0.25% catalyst loading, in this system, equates to approximately £1 of [Rh(C_2_H_4_)Cl]_2_ per 1 g of product.^44^

The efficiency of this procedure belies the challenging nature of the installation of heteroaromatic groups via asymmetric SMC. The demonstration of this methodology on a scale relevant to industry will hopefully enable the modular synthesis of new sp^3^-dense, enantio-enriched compounds to access new libraries of three-dimensional building blocks, aligning with the knowledge that sp^3^-rich molecules are preeminent as successful drug candidates. It is our hope that this work will highlight the potential applicability of these methods to process scale synthesis.

## Experimental Section

### Procedure for Suzuki–Miyaura Coupling on a 100 g Scale

#### Flask A

[Rh(C_2_H_4_)Cl]_2_ (0.56 g, 1.4 mmol, 0.25 mol %) and (*S*)-Segphos (2.11 g, 3.5 mmol, 0.6 mol %) were added to a 250 mL flame-dried flask equipped with a magnetic stir bar. The flask was put under reduced pressure for 5 min and then back-filled with argon. This was repeated once more before THF (153 mL) was added and the flask was stirred at room temperature for 30 min.

#### Flask B

Meanwhile, allyl chloride rac-**2** (100 g, 0.57 mol, 1 equiv) and furan-2-boronic acid pinacol ester **6** (279 g, 1.42 mol 2.5 equiv) were added to a flame-dried 2 L two-neck RBF equipped with a magnetic stir bar, thermometer, and argon balloon. THF (575 mL) and deionized water (75 mL) were added to the flask via a cannula. The flask was placed in an ice bath until the temperature stabilized. CsOH (100 mL, 1.14 mol, 2 equiv) was added via a cannula.

After the temperature of flask B stabilized after the addition of CsOH, and 30 min had passed after solvent addition to flask A, the Rh-ligand containing solution was transferred to flask B via a cannula. After 5 min from the start of the catalyst addition, the reaction flask was placed in a 20 °C water bath.

Upon completion, the reaction mixture was filtered through a celite plug to remove CsCl, and the reaction flask rinsed with Et_2_O (∼50 mL), before being concentrated under vacuum. The resulting brown-red oil was dissolved in THF (300 mL) before 3 M NaOH (200 mL, 3 equiv) was added and the mixture stirred at 40 °C for 1 h. The organic and aqueous layers were partitioned before the organic material was washed with water until no pinacol could be detected by ^1^H NMR (10 × 200 mL) and then concentrated under vacuum. The crude product was purified by vacuum distillation (0.5 mbar, 120 °C) to yield allyl furan **5** as a colorless oil (106.7 g, 90% yield, 98% ee).

See the SI for further details regarding the monitoring of the reaction and characterization data.

## Supporting Information Available

Detailed descriptions of substrate synthesis and set up rhodium-catalyzed reaction; NMR spectra for complexation studies; and graphs of reaction kinetic data and temperature studies (PDF)
