# Supplementary material for: Scale-Up of a Rh-Catalyzed Asymmetric sp3–sp2 Suzuki–Miyaura-Type Reaction
Source: Org Process Res Dev. 2022 Nov 2;26(11):3153–60. doi: 10.1021/acs.oprd.2c00268 (PMC9680029; doi:10.1021/acs.oprd.2c00268)
Supplement: Supplementary file 1 — op2c00268_si_001.pdf [file op2c00268_si_001.pdf]

Scale-up of a Rh-Catalyzed Asymmetric sp^3-sp^2 Suzuki-Miyaura Type Reaction

Laura Cunningham[1], Mireia Sidera Portela[2] and Stephen P. Fletcher[1]*

[1] *Department of Chemistry, Chemistry Research Laboratory, University of Oxford, Oxford, OX1, 3TA UK*

[2] *Vertex Pharmaceuticals (Europe) Ltd, Abingdon, OX14 4RY, UK*

Supplementary Information

Table of contents:

1. Supplementary Figures
   1.1 ^{31}P NMR spectra of complexation studies……………………………………………….. 2
   1.2 Reaction kinetics and temperature studies……………………………………………….. 6
2. Experimental procedures……………………………………………………………………..
   2.1 General Methods…………………………………………………………………………. 9
   2.2 Synthesis of allyl chloride (rac)-**2**………………………………………………………….. 10
   2.3 Procedure for Rh-catalysed SMC………………………………………………………... 10
3. NMR Spectra………………………………………………………………………………… 11
4. SFC Traces…………………………………………………………………………………... 12
5. References…………………………………………………………………………………… 12

1.1 ^{31}P NMR spectra of complexation studies

General procedure for the preparation of NMR samples for complexation studies

Rhodium (0.01 mmol, 1 equiv) and (*S*)-Segphos (0.024 mmol, 2.4 equiv) were weighed and added to a 5 mL flame dried flask equipped with a magnetic stir bar. The flask was put under reduced pressure for 5 minutes, then back-filled with argon. This was repeated once more, before THF (2 mL) was added and the flask was stirred at room temperature for 30 minutes, at which point a sample was removed for NMR analysis.

Preparation of the NMR samples involves:

1. Pre-flush an NMR tube by placing an argon balloon equipped with a long needle in the tube for five minutes.
2. Purge a syringe three times with argon, then extract 0.4 mL of the complexation solution.
3. Dispense the 0.4mL of solution into the NMR tube.
4. Cap NMR tube is immediately and parafilmed, and NMR is submitted within 5 minutes. ^{31}P NMR run time is approximately 16 minutes.

[Rh(cod)OH]$_2$ + (*S*)-Segphos $\xrightarrow[60\ ^{o}C]{THF}$ NMR after 30 minutes

1 equiv. 2.5 equiv.

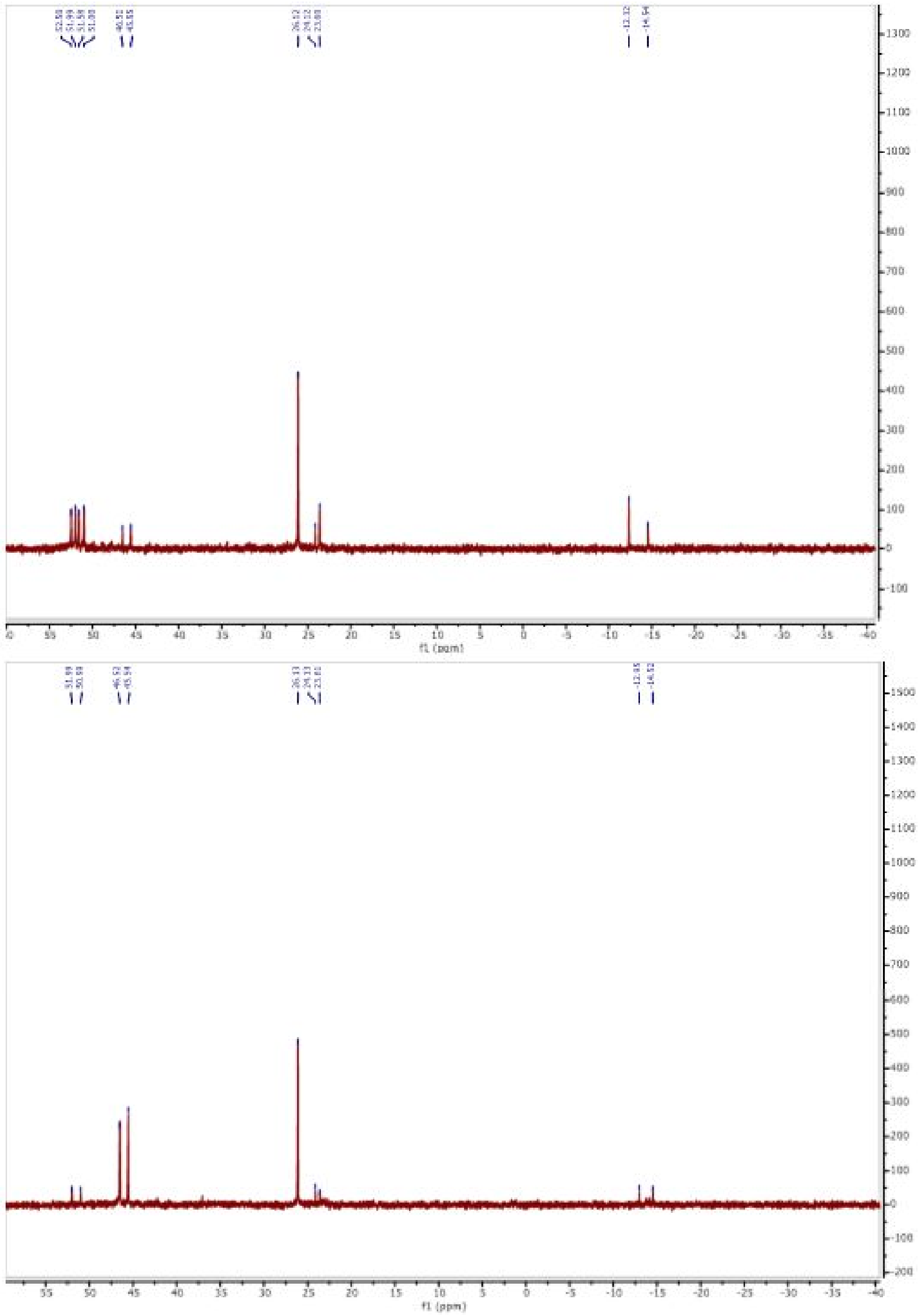

Figure S1. Two selected examples of forming the catalyst complex which were carried out simultaneously. ^{31}P NMR spectroscopic analysis of the reaction of [Rh(cod)OH]$_2$ and (*S*)-Segphos at 60 ºC in THF after 30 min. At least three phosphine-rhodium complex species observed (45-55ppm) in addition to uncomplexed ligand (12ppm), ligand mono-oxide (14ppm and 23ppm) and ligand oxide (26ppm).

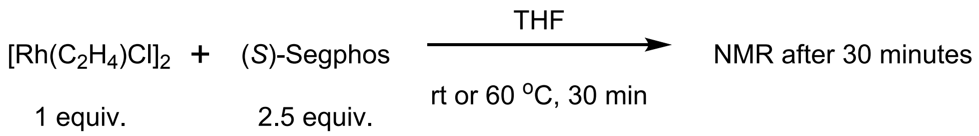

2. A

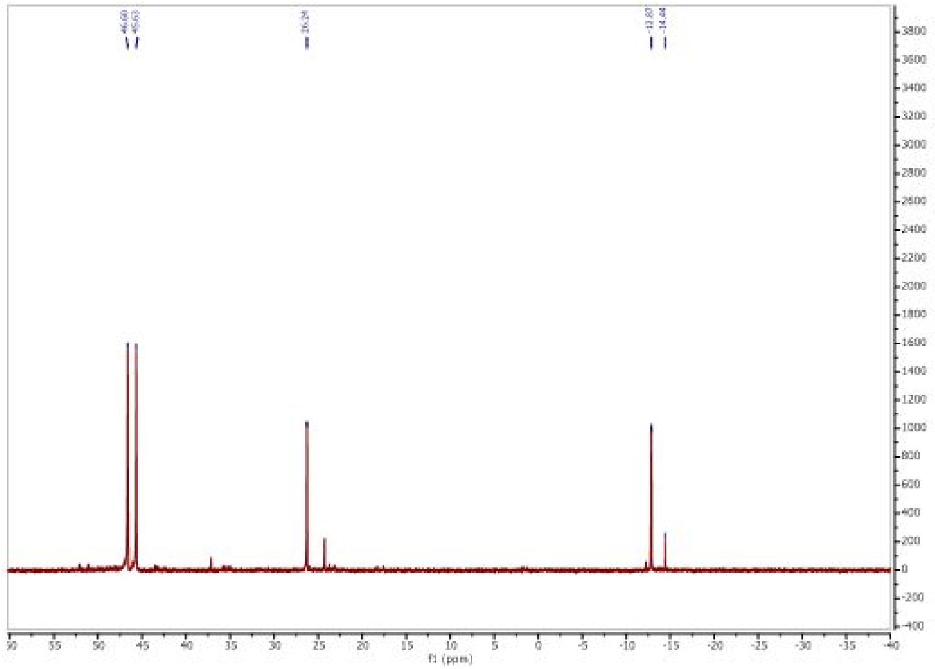

2. B

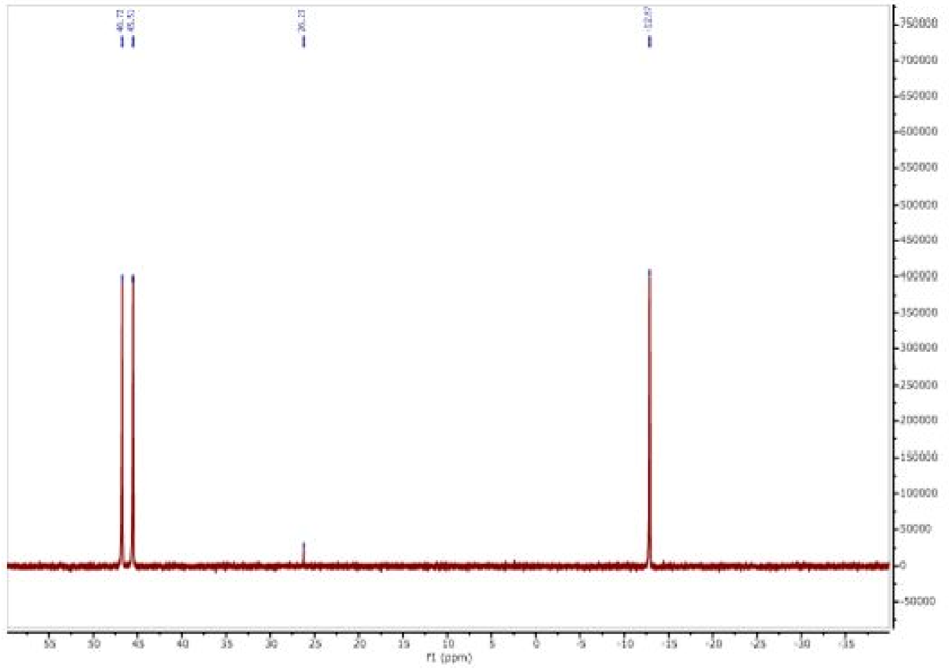

Figure S2. ^{31}P NMR spectroscopy of the reaction between $[Rh(C_2H_4)Cl]_2$ and (*S*)-Segphos in THF after 30 min at room temperature. One major rhodium-ligand complex is observed (doublet 45.5ppm) alongside unreacted ligand (12ppm) and ligand oxide (26ppm). A) Reaction at 60 ºC gave some minor impurities. B) A cleaner reaction is observed at rt.

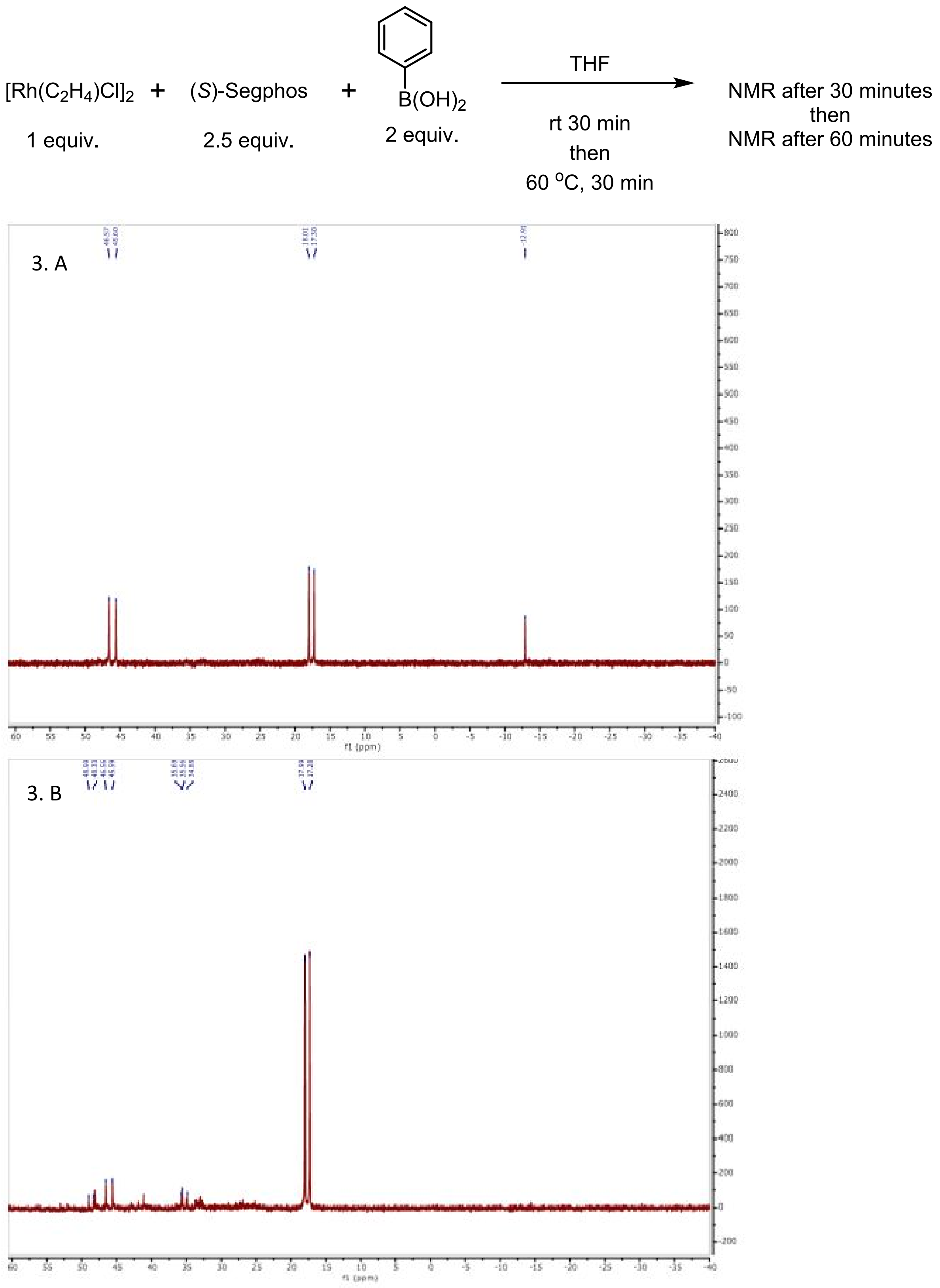

Figure S3. ^{31}P NMR spectroscopic analysis of the reaction of $[Rh(C_2H_4)Cl]_2$, (*S*)-Segphos and phenyl-boronic acid in THF after 30 min. Two major rhodium-ligand species are formed: ligated rhodium: doublet 45.5ppm, transmetallated complex: doublet at 17ppm, Segphos at -12ppm. A) Solution stirred at 25 ºC after 30 min B) Solution A heated to 60 ºC for 30 min.

1.2 Reaction kinetics and temperature studies

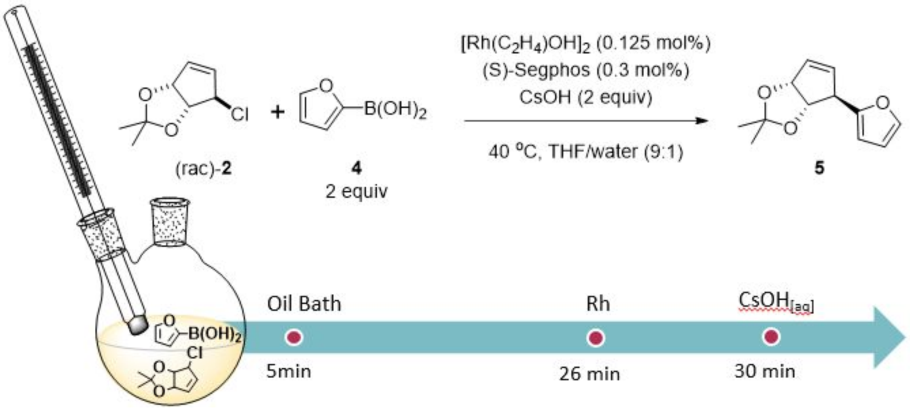

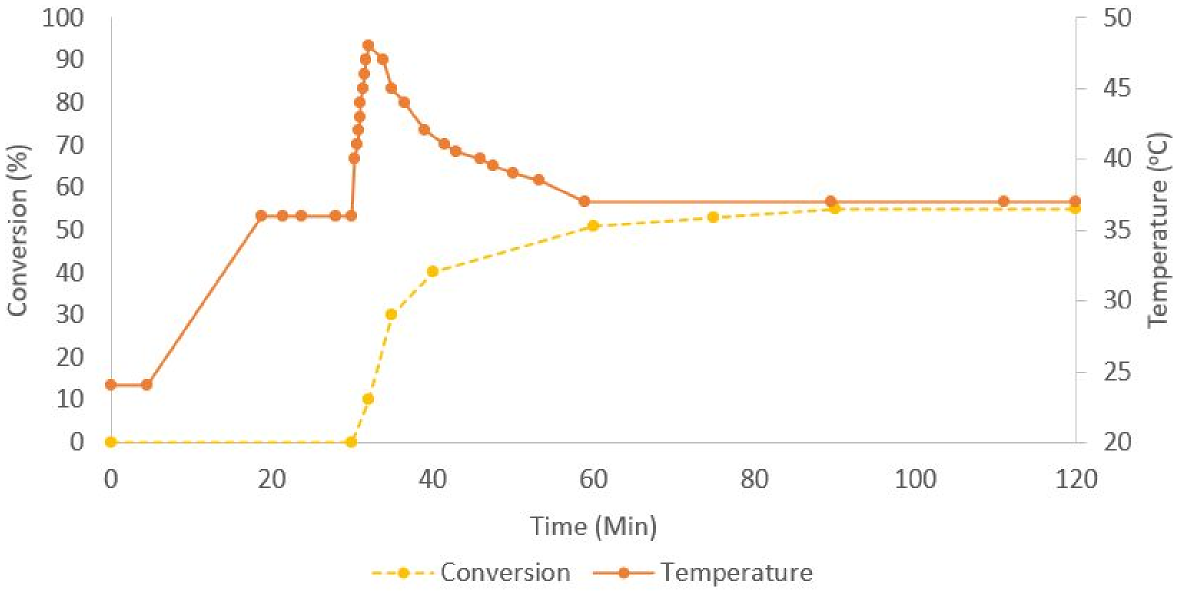

Figure S4. Internal temperature of SMC reaction over time, plotted with % conversion for a multigram test scale reaction. Allyl chloride (2 g, 12 mmol), boronic acid (2 equiv) and THF/water were combined at room temperature (t_0) and then warmed in a 40 °C oil bath after 5 min. Rh-L complex (0.125 mol% $[Rh(C_2H_4)Cl]_2$) added at t=26 min, CsOH added at t=30 min. The temperature spike of ~12 °C was seen immediately after the addition of base.

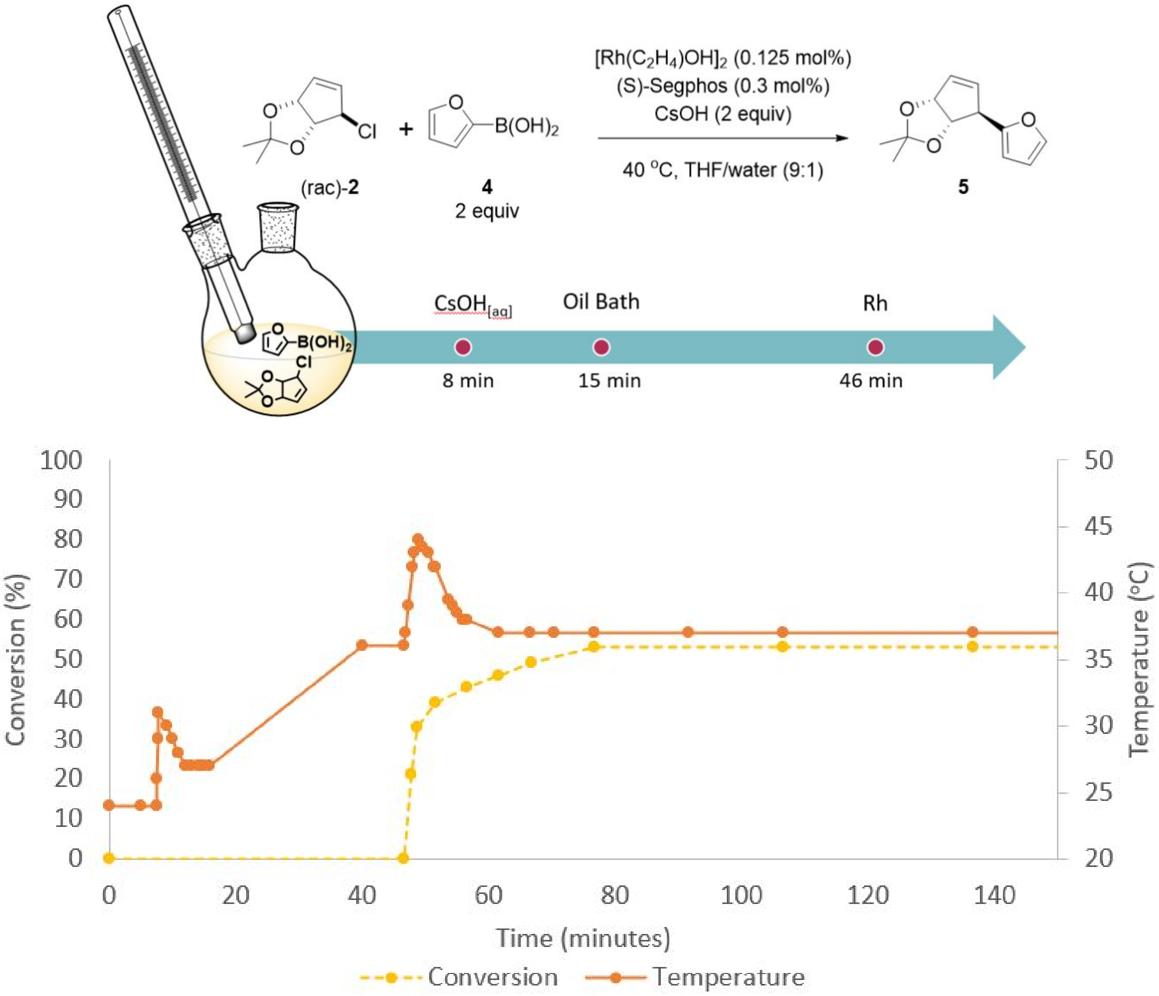

Figure S5. Internal temperature of SMC reaction over time, plotted with % conversion for a multigram test scale reaction. Allyl chloride (2 g, 12 mmol), boronic acid (2 equiv) and THF/water were combined at room temperature (t_0). CsOH added at t=8 min, reaction placed in a 40 ºC oil bath at t=15 min, Rh-L complex (0.125% $[Rh(C_2H_4)Cl]_2$) added at t=46 min. Temperature spikes of 7 ºC recorded upon addition of CsOH, and 8 ºC upon addition of rhodium complex.

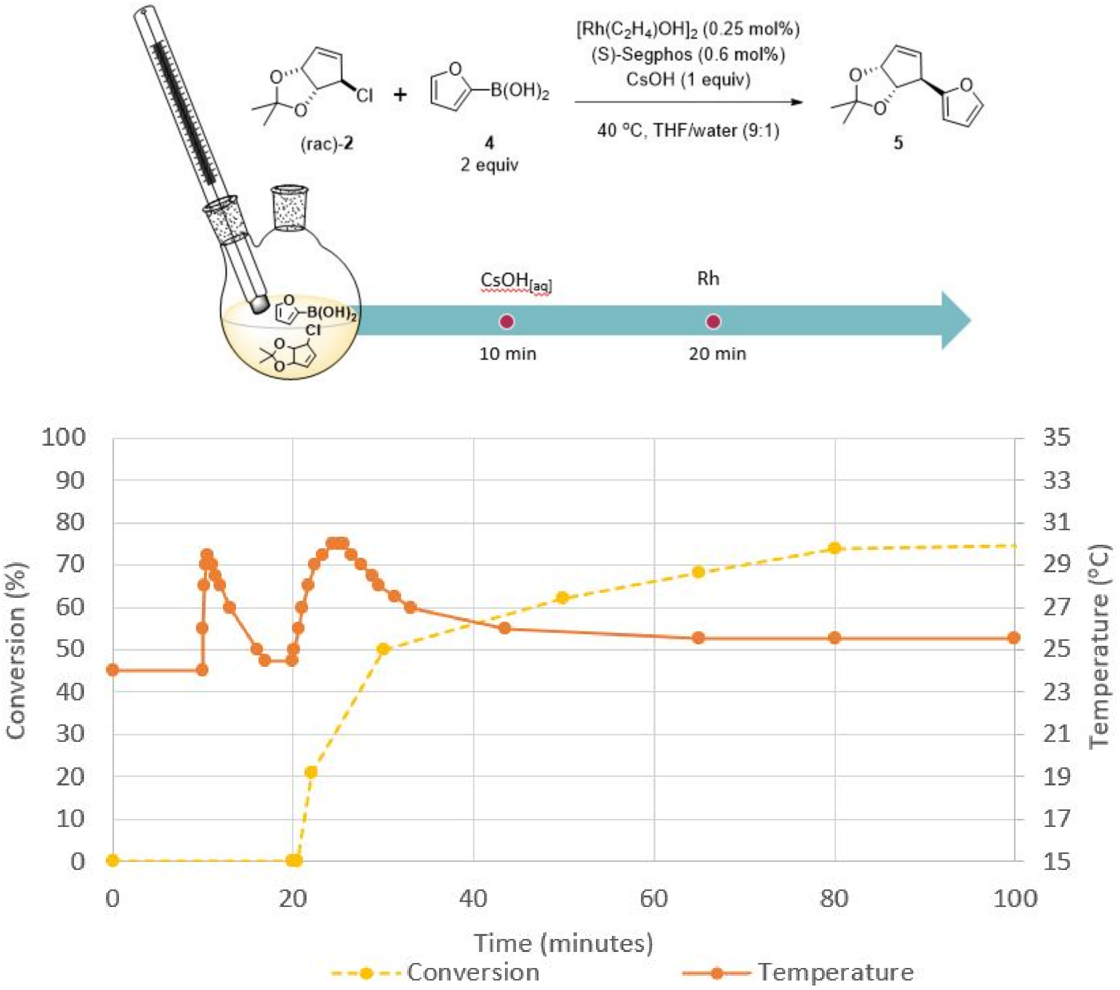

Figure S6. Internal reaction temperature over time of catalysis carried out at room temperature. Allyl chloride (2 g, 12 mmol), boronic acid (2 equiv) and solvent were combined at room temperature (t_0). CsOH added at t=10 min, Rh-L complex (0.25% $[Rh(C_2H_4)Cl]_2$) added at t=20 min. Temperature spikes of 5 ºC recorded upon addition of CsOH, and 5 ºC upon addition of rhodium complex.

Experimental procedures

2.1 General Methods

Procedures using oxygen and/or moisture-sensitive materials were performed with anhydrous solvents (vide infra) under an atmosphere of anhydrous argon in flame-dried flasks, using standard Schlenk techniques. Analytical thin-layer chromatography was performed on precoated glass-backed plates (Silica Gel 60 F254; Merck) and visualised using a combination of UV light (254 nm) and aqueous basic potassium permanganate stain. Flash column chromatography was carried out using Merck 60 Å silica gel.

Nuclear magnetic resonance (NMR) spectroscopy measurements were carried out at room temperature. ^{1}H NMR, ^{13}C NMR, COSY, and HSQC were carried out using Bruker AVG-400 (400/100 MHz) and AVH-400 (400/100 MHz) spectrometers. Chemical shifts (δ) are reported in ppm relative to the residual solvent peak with corresponding coupling constants (*J*) in Hertz (Hz) and multiplicities (s: singlet, d: doublet, t: triplet, q: quartet, m: multiplet)

Infrared (IR, neat or thin film) spectroscopy was carried out on a Bruker Tensor 27 FT–IR spectrometer within internal calibration range of 4000 – 600 cm^{-1}. The samples are reported as absorption maxima in cm^{-1}.

Chiral SFC (supercritical fluid chromatography) separations were conducted on a Waters Acquity UPC2 system using Waters Empower software. Chiralpak® columns (150×3 mm, particle size 3 μm) were used as specified in the text. Solvents used were of HPLC grade (Fisher Scientific, Sigma Aldrich or Rathburn).

High Resolution Mass spectra were carried out by internal service at the University of Oxford. Electron spray ionisation (ESI+) were recorded on a Thermo Exactive with an orbitrap ion analyser.

Commercially available reagents and ligands were purchased from Sigma Aldrich, Alfa Aesar, Acros Organics, Fluorochem and Strem Chemicals and unless otherwise stated were used without further purification. Dry and deuterated solvents were purchased from Sigma Aldrich.

2.2 Synthesis of allyl chloride (rac)-2

Allyl chloride (rac)-**2** was prepared by modification of procedures previously described.[1,2]

2.3 Procedure for Rh-catalysed SMC

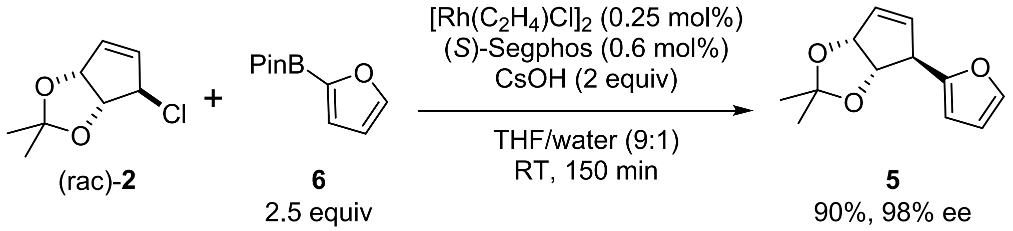

Flask A. $[Rh(C_2H_4)Cl]_2$ (0.56 g, 1.4 mmol, 0.25 mol%) and (*S*)-Segphos (2.11 g, 3.5 mmol, 0.6 mol%) were weighed and added to a 250 mL flame dried flask equipped with a magnetic stir bar. The flask was put under reduced pressure for 5 minutes, then back-filled with argon. This was repeated once more, before THF (153 mL) was added and the flask was stirred at room temperature for 30 minutes.

Flask B. Meanwhile, allyl chloride rac-**2** (100 g, 0.57 mol, 1 equiv.) and furan-2-boronic acid pinacol ester **6** (279 g, 1.42 mol 2.5 equiv) were added to a flame dried 2 L 2-neck RBF equipped with a magnetic stir bar, thermometer and argon balloon. THF (575 mL) and deionised water (75 mL) were added to the flask *via* cannula. The flask was placed in an ice bath until the temperature stabilised. CsOH (100 mL, 1.14 mol, 2 equiv) was added *via* cannula.

After the temperature of flask B stabilised after the addition of CsOH, and 30 minutes had passed after solvent addition to flask A, the Rh-ligand containing solution was transferred to flask B *via* canula. After 5 min from the start of the catalyst addition, the reaction flask was placed in a 20 ºC water bath. The reaction progress was monitored by quantitative ^{1}H NMR spectroscopy, with 0.2 mL aliquots being removed and diluted with 0.5 mL $CDCl_3$ every 5 minutes for the first hour, and every 10 minutes thereafter.

Complete conversion was observed after 90 min, and the reaction mixture was filtered through a celite plug to remove CsCl, and the reaction flask rinsed with Et_2O (~50mL), before being concentrated under vacuum. The resulting brown-red oil was dissolved in THF (300 mL) before 3 M NaOH (200 mL, 3 equiv.) was added and the mixture stirred at 40 ºC for 1 h. The organic and aqueous layers were partitioned before the organic material was washed with water until no pinacol could be detected by ^{1}H NMR (10 x 200 mL) and then concentrated under vacuum. The crude product was purified by vacuum distillation (0.5 mbar, 120 ºC) to yield allyl furan **5**as a colourless oil (106.7 g, 90% yield, 98% ee).

^{1}H NMR ($CDCl_3$, 400 MHz): δ (ppm) 7.33 (dd, *J* = 1.9, 0.9 Hz, 1H, 6.28 (dd, *J* = 3.2, 1.9 Hz, 1H), 6.00 (dt, *J* = 3.2, 0.9 Hz, 1H), 5.97 (dt, *J* = 5.7, 1.9 Hz, 1H), 5.89 (ddt, *J* = 5.7, 2.4, 0.9 Hz, 1H), 5.27 (dq, *J* = 5.9, 1.5 Hz, 1H), 4.68 (d, *J* = 5.7, 1H), 4.08 (dq, *J* = 2.4, 1.3 Hz, 1H), 1.46 (s, 3H), 1.35 (s, 3H). **^{13}C NMR** (101 MHz, $CDCl_3$): δ (ppm) 154.6, 142.0, 132.9, 132.6, 110.8, 110.4, 105.6, 85.3, 83.2, 51.3, 27.6, 25.9. **IR** (λ_{max}/cm^{-1}) 2983, 2935, 2360, 2342, 1506, 1458, 1371, 1333, 1282, 1251, 1211, 1159, 1107, 1070, 1050, 1014, 952, 920, 871, 806, 789. **HRMS** (ESI) m/z: $[M + H]^+$ Calc for $C_{12}H_{15}O_3$ 207.1016; Found 207.1019. **$[\alpha]^{25}_D$** = -216.5 (c = 1.0, $CHCl_3$).

SFC Conditions: Chiralpak IG; 1500 psi, 30 °C; flow: 1.5 mL/min; from 1% to 30% MeOH in 5 min; (major enantiomer t_R = 1.68 min; minor enantiomer t_R = 2.13 min), 98% ee.

3. NMR Spectra

^{1}H NMR spectrum of **5**

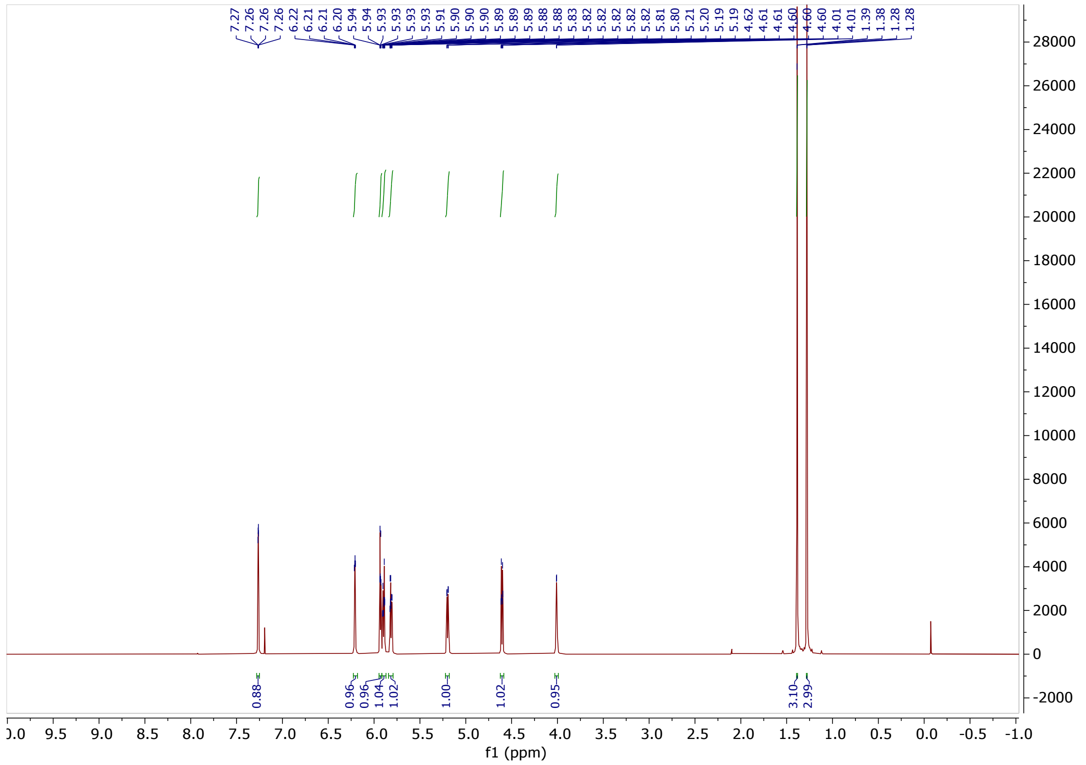

^{13}C NMR spectrum of **5**

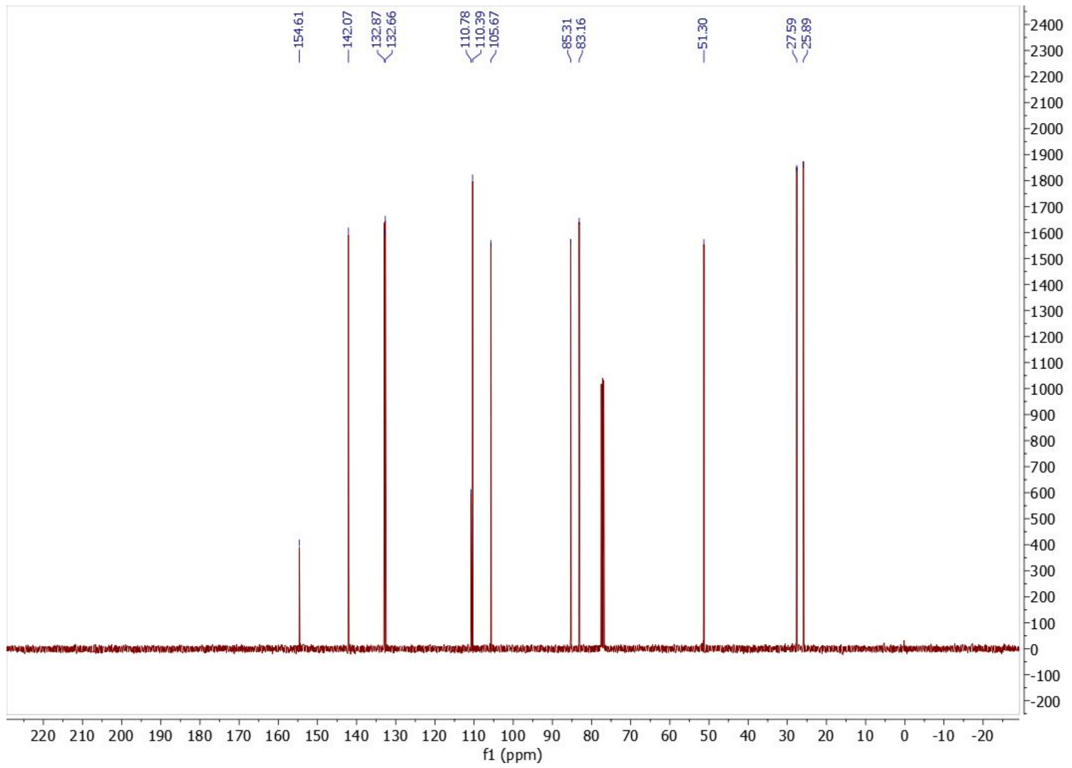

4. SFC Traces

(rac)-**5**

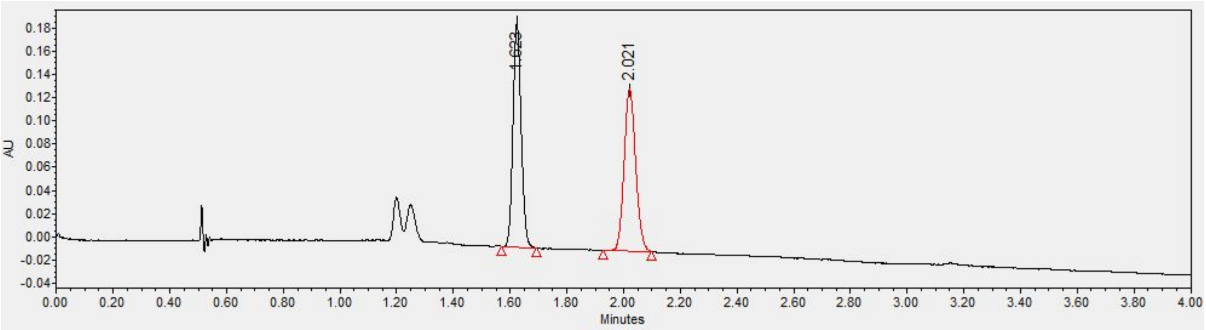

(ent)-**5**

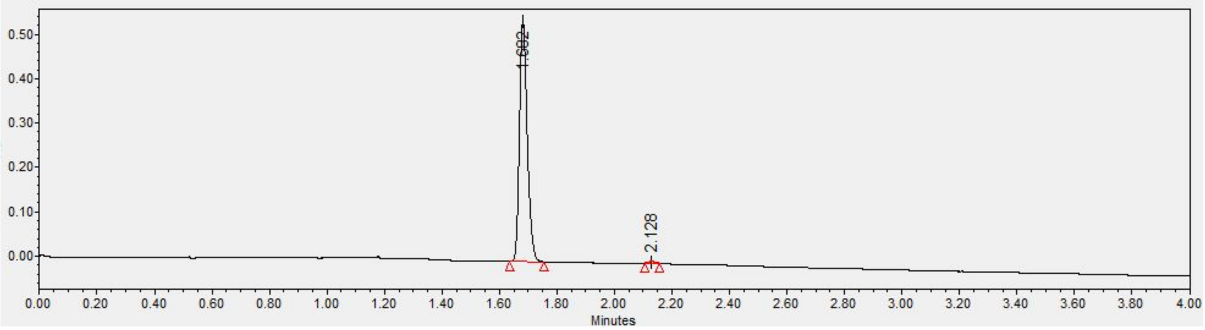

	Name	Retention Time (min)	Area (µV*sec)	% Area	Height (µV)
1		1.682	1019153	99.38	539807
2		2.128	6353	0.62	3569

5. References

(1) Goetzke, F. W.; Mortimore, M.; Fletcher, S. P. Enantio- and Diastereoselective Suzuki–Miyaura Coupling with Racemic Bicycles. *Angew. Chemie Int. Ed.* **2019**, *58*, 12128–12132.

(2) Kučera, R.; Goetzke, F. W.; Fletcher, S. P. An Asymmetric Suzuki-Miyaura Approach to Prostaglandins: Synthesis of Tafluprost. *Org. Lett.* **2020**, *22*, 2991–2994.
